# A Multivariate Approach for the Determination of the Optimal Mixing Ratio of the Non-Strong Interacting Co-Amorphous System Carvedilol-Tryptophan

**DOI:** 10.3390/molecules26040801

**Published:** 2021-02-04

**Authors:** Rong Di, Jingwen Liu, Holger Grohganz, Thomas Rades

**Affiliations:** Department of Pharmacy, University of Copenhagen, DK-2100 Copenhagen, Denmark; rong.di@sund.ku.dk (R.D.); jingwen.liu@sund.ku.dk (J.L.); thomas.rades@sund.ku.dk (T.R.)

**Keywords:** co-amorphous systems, optimal mixing ratio, glass transition temperature, principal component analysis

## Abstract

Converting crystalline compounds into co-amorphous systems is an effective way to improve the solubility of poorly water-soluble drugs. It is, however, of critical importance for the physical stability of co-amorphous systems to find the optimal mixing ratio of the drug with the co-former. In this study, a novel approach for this challenge is presented, exemplified with the co-amorphous system carvedilol–tryptophan (CAR–TRP). Following X-ray powder diffraction (XRPD) and differential scanning calorimetry (DSC) of the ball-milled samples to confirm their amorphous form, Fourier-transform infrared spectroscopy (FTIR) and principal component analysis (PCA) were applied to investigate intermolecular interactions. A clear deviation from a purely additive spectrum of CAR and TRP was visualized in the PCA score plot, with a maximum at around 30% drug (mol/mol). This deviation was attributed to hydrogen bonds of CAR with TRP ether groups. The sample containing 30% drug (mol/mol) was also the most stable sample during a stability test. Using the combination of FTIR with PCA is an effective approach to investigate the optimal mixing ratio of non-strong interacting co-amorphous systems.

## 1. Introduction

Most low-molecular weight drug candidates today are poorly water-soluble, causing insufficient drug dissolution and subsequently a poor bioavailability after oral administration [1,2]. These challenges can amount to a significant problem in pharmaceutical development. One of the commonly used methods to address this problem is using the amorphous (molecularly disordered) solid form of the drug, due to its increased apparent solubility and improved dissolution compared to the usually used crystalline drug forms [1,3]. However, amorphization may lead to challenges due to the thermodynamic instability of amorphous forms, which can lead to recrystallization, e.g., during formulation development, manufacture, storage or administration [1,3]. One approach to avoid (or at least to significantly slow down) crystallization is introducing a co-former to stabilize the drug [4]. The co-former (which is another low molecular weight, initially crystalline compound) can form a homogeneous amorphous mixture on the molecular level with the drug through intermolecular interactions, potentially additionally causing an increase in the glass transition temperature (T_g_) of the co-amorphous system compared to the pure amorphous drug; both processes stabilize the amorphous drug [5,6,7]. Compared to other approaches, such as the development of drug–polymer amorphous solid dispersions (ASDs), co-amorphous systems often show higher stability and almost always lead to a lower excipient usage (allowing for higher drug doses to be administered) [8]. However, co-amorphous systems can still fail in long-term physical stabilization in some cases, especially if an excess of drug or co-former is used in the co-amorphous system [9]. The excess component can lead to phase separation and recrystallization, and thus poses a significant risk to pharmaceutical development [10]. Therefore, investigating the optimal mixing ratio between a drug and a co-former is highly significant for creating a long-term stable co-amorphous system.

The co-former can interact with the drug through different kinds of molecular interactions which can be divided into non-strong interactions (such as hydrogen bond formation and π–π interactions) and strong interactions (such as ionic interactions) [11,12,13,14]. A method of detecting the optimal mixing ratio of strongly interacting co-amorphous systems was suggested by Liu et al. and a method for non-strongly interacting co-amorphous systems was investigated by Kissi et al., but the latter method was experimentally demanding and not widely accessible [6,15]. Therefore, it would be beneficial if another, less complex, method could be developed. In this study, a new method was developed to determine the optimal mixing ratio of drug to co-former in non-strongly interacting co-amorphous systems. The method uses the application of multivariate analysis to the Fourier-transform infrared spectroscopy (FTIR) data of various co-amorphous samples. Carvedilol (CAR) and tryptophan (TRP) were used in the current study as model drug and co-former, respectively.

## 2. Results and Discussion

### 2.1. Investigation of the Molecular Interaction of the Co-Amorphous Systems by Using the Gordon–Taylor Equation

CAR–TRP samples, containing 10–90% drug (mol/mol), were subjected to ball milling and characterized by X-ray powder diffraction (XRPD) and differential scanning calorimetry (DSC) to determine the solid form and the miscibility of the samples, respectively. All CAR–TRP samples were converted to fully co-amorphous systems (results can be found in the supplementary information). These preparation and characterization methods have already been used by Kissi et al., who provide a more detailed explanation [6].

**Figure 1:**
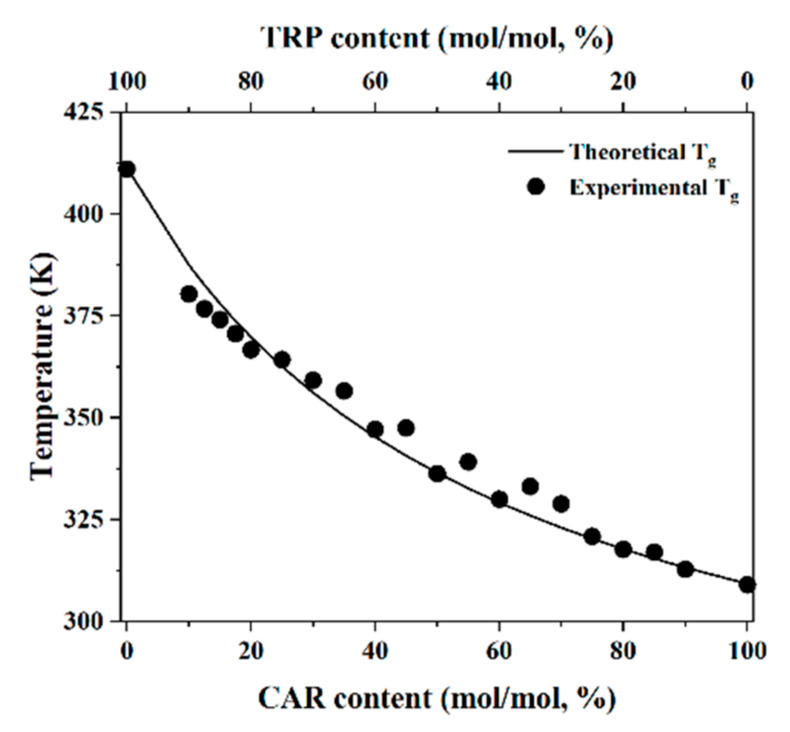
Experimental and theoretical T_g_ values of co-amorphous carvedilol–tryptophan (CAR–TRP) samples with different drug contents.

The Gordon–Taylor equation was then applied to investigate the optimal mixing ratio. Application of this equation has already been shown to be an effective way of finding the optimal mixing ratios in strongly interacting co-amorphous systems, as there are deviations between the experimental T_g_ values and the theoretical T_g_ values calculated from this equation. The Gordon–Taylor equation assumes a homogenous mixture without any interactions, thus the existence of molecular interactions causes a deviation of the experimental T_g_ from the theoretical (Gordon–Taylor equation) T_g_ [5,6,16,17,18]. Therefore, the Gordon–Taylor equation was also used to investigate the optimal mixing ratio of the non-strongly interacting co-amorphous CAR–TRP systems. The experimental T_g_ values were obtained from the DSC measurements and the theoretical T_g_ values were calculated with the Gordon–Taylor equation. By comparison, no large deviations were found between theoretical and experimental T_g_ values (Figure 1) which showed that no strong molecular interactions could be found between CAR and TRP. In other words, the interaction between CAR and TRP is too weak to show a difference between the experimental and theoretical T_g_ values. Thus, the Gordon–Taylor equation could not be used to find an optimal mixing ratio in this non-strongly interacting co-amorphous system. Similar T_g_ values were also obtained in a study by Kissi et al. [6].

### 2.2. Investigation of Intermolecular Interaction of the Co-Amorphous Systems by Fourier-Transform Infrared Spectroscopy

**Figure 2:**
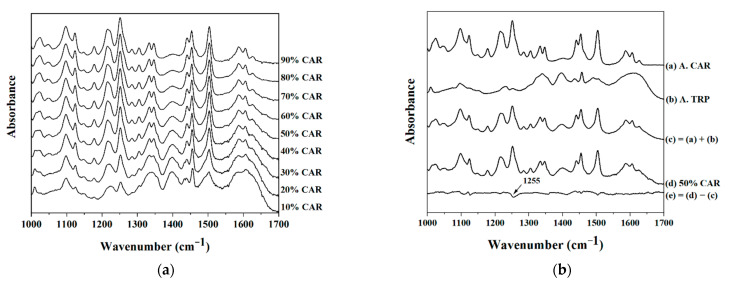
(**a**) Fourier-transform infrared spectroscopy (FTIR) spectra (after standard normal variate (SNV) correction) of co-amorphous CAR–TRP samples with different drug contents (10–90% (mol/mol)). (**b**) FTIR spectra of amorphous CAR, amorphous TRP, amorphous CAR combined with amorphous TRP (combination spectrum), co-amorphous CAR–TRP sample (50% drug content) and 50% CAR–TRP subtracted from the combination spectrum.

Next, FTIR was used to investigate molecular interactions within the CAR–TRP systems. First the spectral range of 1000–1700 cm^−1^ was chosen to be analyzed since it includes information about changes in the aromatic ring systems (1100–1500 cm^−1^), as well as hydrogen-bonded carboxylic acids (1700 cm^−1^) and amides (1600 cm^−1^) [16,19]. Then, standard normal variate (SNV) correction was applied before analyzing the FTIR data. The results after SNV correction are shown below (Figure 2a).

An N–H bending vibration (1503 cm^−1^), C–N stretching vibration (1214 cm^−1^) and C–O stretching vibration (1255 cm^−1^) could be found at all mixing ratios. Differences between the different CAR–TRP samples, however, were not obvious. Therefore, the spectra of pure amorphous CAR and pure amorphous TRP were determined and then combined at equal weighting. This combination spectrum was then compared with the spectrum of the CAR–TRP sample (50% drug content) by subtracting the CAR–TRP sample (50% drug content) spectrum from the combination spectrum (Figure 2b). Thus, a new spectrum which showed the difference between an amorphous mixture (no interactions) and the co-amorphous sample (possibly weakly interacting) was obtained. The subtracted spectrum (Figure 2b, spectrum (e)) was relatively flat and indicates the absence of strong interactions between CAR and TRP in the co-amorphous samples. However, a slight “dip” can be found in the graph after the subtraction, at 1255 cm^−1^. Since 1255 cm^−1^ represents the stretching vibration of ether groups (from CAR), and TRP has several functional groups allowing it to form hydrogen bonds [16,20], hydrogen bonds may be formed between the ether group (hydrogen bond acceptor) of CAR and a hydrogen bond donating group of TRP. This newly established hydrogen bond could contribute to a stabilizing effect in the co-amorphous system.

CAR has other functional groups that can form hydrogen bonds, such as methoxy and amino groups [21]. The reason that CAR formed a hydrogen bond with TRP via the ether group may have been because amorphous CAR forms hydrogen bonds with other CAR molecules before forming hydrogen bonds with TRP. Therefore, most of the functional groups that were able to form hydrogen bonds with TRP were already occupied and CAR could only accept hydrogen bonds via the ether group.

### 2.3. Principal Component Analysis of FTIR Data

**Figure 3:**
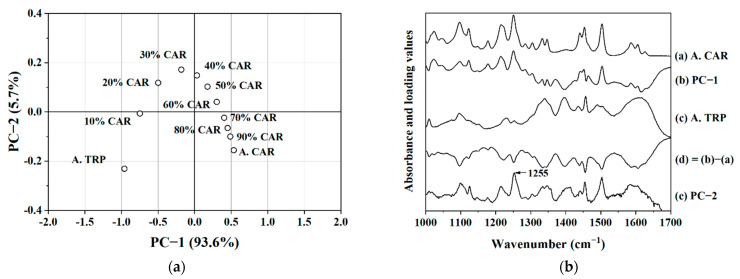
(**a**) Score plot of the principal component analysis (PCA) on the FTIR data. PC-1 (explained 93.6% of the variation) was plotted against PC-2 (explained 5.7% of the variation). (**b**) FTIR spectra of amorphous CAR, amorphous TRP, loading plot of PC-1, a graph of amorphous CAR subtracted from the loading plot of PC-1, and loading plot of PC-2. The black arrow shows the highest peak (1255 cm^−1^) in the loading plot.

It is difficult to visually distinguish any differences between the FTIR spectra of samples with different mixing ratios. Even the above outlined subtraction procedure can only provide an indication. Therefore, principal component analysis (PCA) was used to further analyze the FTIR data and the score plot is shown in Figure 3a. In the score plot of the PCA results, the neat amorphous CAR sample had the highest value in the first principal component (PC-1) and the neat amorphous TRP sample had the lowest value. The higher the content of CAR, the higher the value it had in PC-1. This was also verified by the loading of PC-1, which showed a strong resemblance with the FTIR spectrum of CAR. By subtracting the FTIR spectrum of neat amorphous CAR from the loading plot of PC-1, a new graph was obtained (Figure 3b). The new graph looks like an inverse form of the FTIR spectrum of amorphous TRP. This, along with the clear order in the distribution of the samples along PC-1, indicates that PC-1 mainly described the ratio between CAR and TRP in the samples. The content of CAR in the co-amorphous samples made a positive contribution to the PC-1 values and the TRP content made a negative contribution to the PC-1 values. PC-1 explained 93.6% of the variation, which indicates that the major variation in the FTIR spectra was due to the varying ratio of the components.

The findings related to PC-1 were foreseeable and did not indicate any change in interaction pattern. It was therefore of higher interest to investigate PC-2, which explained 5.7% of the variation. If no additional weaker interactions were present, a random distribution of the samples along PC-2 would have been expected. However, it is clearly visible in the score plot that systematic variation occurred with changes in the CAR–TRP ratio. From neat amorphous TRP to CAR–TRP samples containing up to 30% drug, the score values increased and the CAR–TRP sample with 30% drug content obtained the highest score on PC-2. Then, the score values decreased with the further increase of drug content from the CAR–TRP sample containing 40% CAR to the neat amorphous CAR sample. The pattern of the score values of PC-2 can be attributed to an interaction between CAR and TRP. The loading plot of PC-2 (Figure 3b) shows a strong contribution of the vibration at 1255 cm^−1^. This means the ether groups of CAR were responsible for the highest value of the CAR–TRP sample with 30% drug content in PC-2 and may be indicative of the ether groups being involved in the interaction between CAR–TRP. FTIR and PCA thus indicated that 30% CAR to 70% TRP (mol/mol) may be the optimal mixing ratio. A study performed by Kissi et al. also described a method to find the optimal mixing ratio of this co-amorphous system [6]. The authors investigated the beta relaxation of different mixing ratios of CAR and TRP by dynamic mechanical analysis. Samples with excess TRP showed a similar temperature for the beta relaxation and samples with excess CAR also showed a similar temperature for the beta relaxation but different from that of samples with excess TRP. The temperature for the beta relaxation thus changed significantly at the optimal mixing ratio, which was suggested to be around 40% CAR.

### 2.4. Physical Stability of the Co-Amorphous Systems under Dry Storage Conditions

According to the DSC and FTIR results, 30% CAR–70% TRP (mol/mol) was considered as the optimal mixing ratio for the CAR–TRP co-amorphous systems. To test this assumption, a physical stability test was conducted. During the physical stability test, XRPD was used to characterize the solid state of the co-amorphous samples upon storage. The samples were stored under dry conditions at different temperatures (room temperature and 50 °C).

**Figure 4:**
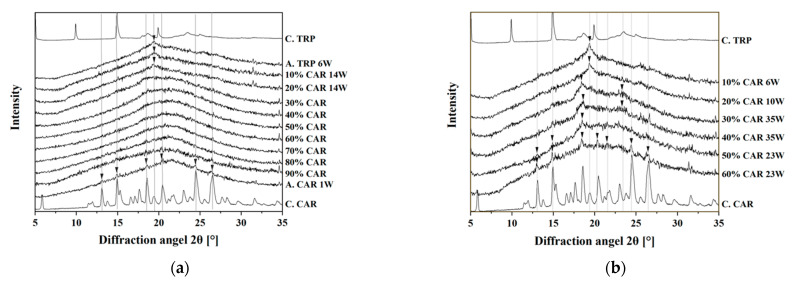
(**a**) X-ray powder diffraction (XRPD) diffractograms of various CAR–TRP samples stored at room temperature, dry condition; (**b**) XRPD diffractograms of various CAR–TRP samples stored at 50 °C, dry condition. The arrows show peaks after recrystallization.

Most samples stored at room temperature were still stable after ten months of storage (Figure 4a). In contrast, pure amorphous CAR and pure amorphous TRP recrystallized after one week and six weeks of storage, respectively. CAR–TRP samples with 10% and 20% CAR recrystallized after 14 weeks. All other samples (with 30–90% CAR) were still stable at the conclusion of the stability study. All samples stored at 50 °C recrystallized during the stability study (Figure 4b). Samples containing 10% CAR recrystallized after six weeks and samples containing 20% CAR recrystallized after ten weeks. Samples with 30% and 40% CAR recrystallized after 35 weeks, whereas samples with 50% and 60% CAR stayed stable for 23 weeks. Hence, samples containing 30% and 40% CAR were the most stable samples. This corresponds to the result of the DSC and FTIR investigations.

## 3. Materials and Methods

### 3.1. Materials

CAR (molecular weight: 406.474 g/mol) was purchased from Cipla Ltd. (Mumbai, India); TRP (204.23 g/mol) was purchased from Sigma-Aldrich (St. Louis, MO, USA).

### 3.2. Methods

#### 3.2.1. Preparation of Co-Amorphous Systems by Ball Milling

The co-amorphous samples were prepared in a mixer mill MM 400 from Retsch GmbH & Co. (Haan, Germany). In total, 1000 mg of CAR and TRP (at various ratios) was weighed and transferred to 25 mL stainless steel jars and ball milled for 90 to 360 min using two stainless steel balls (12 mm diameter) at a frequency of 30 Hz. The mill was placed in a cold environment (around 6 °C). All samples are presented by using molar ratio from 10% to 90% (CAR/TRP).

#### 3.2.2. Characterization of Solid States by XRPD

The solid state of the samples was characterized using an X’Pert PANalytical PRO x-ray diffractometer (PANalytical, Almelo, The Netherlands), using Cu Ka radiation (1.54187 Å) and an acceleration voltage and current of 45 kV and 40 mA, respectively. The samples were placed on a plate and scanned from 5 to 35° 2θ in reflection mode, with a scan rate and scan step of 0.0625° 2θ/s and 0.026° 2θ, respectively. Bragg–Brentano parafocusing geometry was used. The data were collected and analyzed using the software X’Pert Data Collector (PANalytical, Almelo, The Netherlands).

#### 3.2.3. Characterization of *T_g_* by DSC

The *T_g_* of the sample was determined using a Discovery DSC (TA Instruments, New Castle, DE, USA) under a 50 mL/min nitrogen gas flow. Sample powders of 2–5 mg were weighed into Tzero aluminium pans and sealed with Tzero lids. A modulated measuring mode was used in the DSC measurements. First, the samples were kept isothermal at −20.00 °C for 5 min and then heated up at an average rate of 2.00 °C/min to 160.00 °C, with amplitude and period of 0.2120 °C and 40 s, respectively. Data were collected and analyzed by the Trios software, version 5.0.0.44608 (TA Instruments, New Castle, DE, USA). The *T_g_* was determined from the reversing heat flow signal. The recrystallization and melting events were observed from the total heat flow signal.

#### 3.2.4. Calculation of Theoretical *T_g_* with the Gordon–Taylor Equation

Theoretical *T_g_* was calculated with the Gordon–Taylor equation:

(1) $$T_{g12}=\frac{\omega_{1}\times T_{g1}+K\times \omega_{2}\times T_{g2}}{\omega_{1}+K\times \omega_{2}}$$

where *T_g_*_12_ and *T_g_*_1_ are the *T_g_* of the co-amorphous samples and the components and *ω_i_* is the mass fraction of the component *i*. *K* is a constant and can be calculated by the equation:

(2) $$K=\frac{T_{g1}\times \rho_{1}}{T_{g2}\times \rho_{2}}$$

where *ρ*_1_ and *ρ*_2_ are the densities of the two components (*ρ_amorphous−CAR_* = 1.24 g/cm^3^) [22]. No information for the amorphous TRP was available in the literature, so it was calculated by the following equation:

(3) $$\rho_{amorphous-TRP}=\frac{\rho_{amorphous-CAR}}{\rho_{crystalline-CAR}}\times \rho_{crystalline-TRP}$$

where *ρ_crystalline−CAR_* = 1.26 g/cm^3^, *ρ_crystalline−TRP_* = 1.30 g/cm^3^ [22,23].

#### 3.2.5. Investigation of Intermolecular Interactions by FTIR

The FTIR spectra were collected using a Bomem FTIR spectrometer (MB-Series, ABB Bomem Inc., Quebec, QC, Canada). Samples were scanned 64 times at a wavenumber range from 400 to 4000 cm^−1^ with a resolution of 4 cm^−1^. Data was collected by GRAMS/AI software (version 7.0, Thermo Fisher Scientific, Waltham, MA, USA) and analyzed using Origin software (version 9.6.0.172, OriginLab Corporation, Northampton, MA, USA).

#### 3.2.6. Physical Stability

The co-amorphous samples were stored at two different dry conditions (room temperature and 50 °C) to test the physical stability. P_2_O_5_ was used to maintain the dry conditions. XRPD was used to characterize the solid-state forms of the samples.

#### 3.2.7. Multivariate Data Analysis

The spectral range of 1000–1700 cm^−1^ was used for further analysis as it included relevant information, such as changes in aromatic ring systems (1100–1500 cm^−1^), hydrogen-bonded carboxylic acids (1700 cm^−1^) and amides (1600 cm^−1^). Standard normal variate transformation was conducted on the chosen range to reduce the variation due to the physical characteristics of the samples. The obtained preprocess spectra were investigated by PCA using the software Simca 14.1 (Umetrics AB, Umeå, Sweden).

## 4. Conclusions

CAR–TRP co-amorphous systems were used as a model of non-strongly interacting systems to detect their optimal mixing ratio. The co-amorphization of CAR–TRP samples with different mixing ratios can be achieved successfully by ball milling. A melting event (i.e., crystallization upon heating) was found in the DSC thermograms of samples with more than 40% CAR, indicating an excess of CAR in these samples. The FTIR data confirmed a weak interaction between CAR and TRP. CAR and TRP may form hydrogen bonds via the ether group of CAR. PCA on FTIR data indicated that 30% CAR to 70% TRP (mol/mol) may be the optimal mixing ratio and this was confirmed in a physical stability test. The combination of FTIR and PCA may be a practical method to find the optimal mixing ratio in non-strongly interacting co-amorphous system.

## Data Availability

The data presented in this study are available on request from the corresponding author.

## Supplementary Materials

The following are available online, Figure S1: XRPD diffractograms of CAR–TRP samples with different drug contents (10–90% (mol/mol)) after different ball milling times (90–360 min). The grey dashed line shows the halo maximum intensity of TRP. The grey solid line shows the halo maximum intensity of CAR., Figure S2: (a) DSC thermograms (reversing heat flow) of co-amorphous CAR–TRP samples with different drug contents (10–90% (mol/mol)). The black arrows show the Tgs of the different samples. (b) DSC thermograms (total heat flow) of co-amorphous CAR–TRP samples with different drug contents (10–90% (mol/mol)). The grey dashed line shows the Tm of CAR.
