# Supplementary material for: A Multivariate Approach for the Determination of the Optimal Mixing Ratio of the Non-Strong Interacting Co-Amorphous System Carvedilol-Tryptophan
Source: Molecules. 2021 Feb 4;26(4):801. doi: 10.3390/molecules26040801 (PMC7913994; doi:10.3390/molecules26040801)
Supplement: Supplementary file 1 [file molecules-26-00801-s001.pdf]

Supplementary Materials

Figure 1. XRPD diffractograms of CAR-TRP samples with different drug contents (10 – 90% (mol/mol)) after different ball milling times (90 – 360 min). The grey dashed line shows the halo maximum intensity of TRP. The grey solid line shows the halo maximum intensity of CAR.

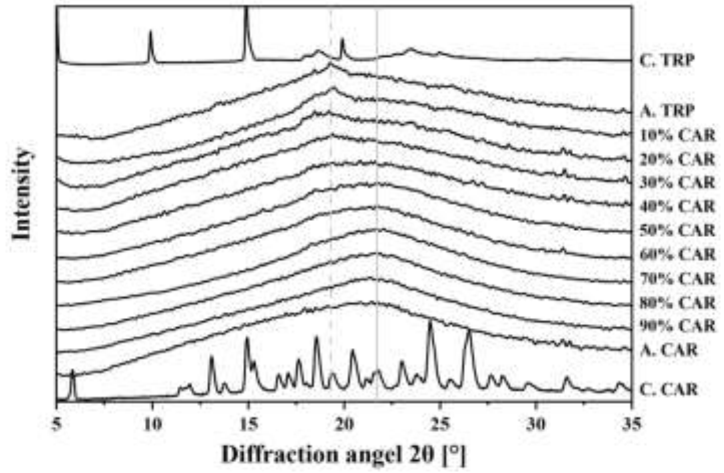

Figure 2. (a) DSC thermograms (reversing heat flow) of co-amorphous CAR-TRP samples with different drug contents (10 – 90% (mol/mol)). The black arrows show the Tgs of the different samples. (b) DSC thermograms (total heat flow) of co-amorphous CAR-TRP samples with different drug contents (10 – 90% (mol/mol)). The grey dashed line shows the Tm of CAR.

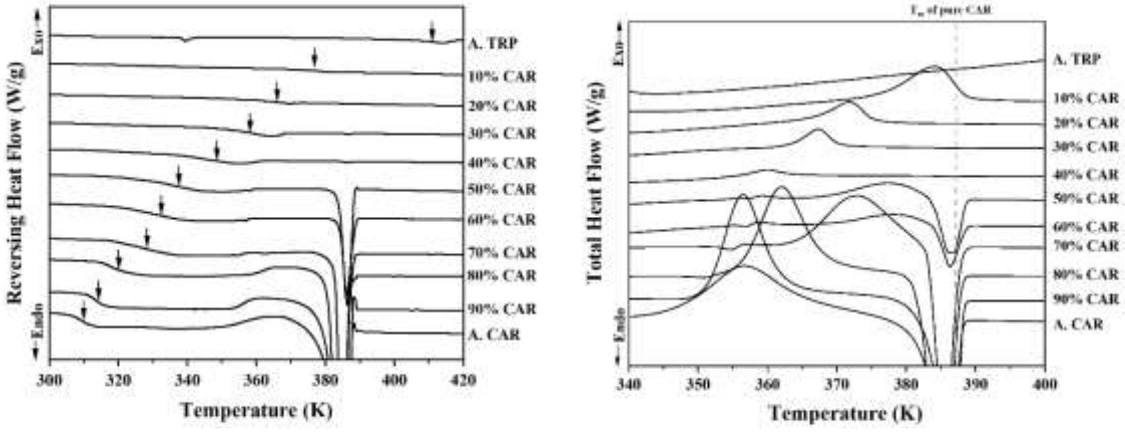

(**a**) (**b**)
